# Compact subsets of autism screening items predict clinical diagnoses with a machine learning analysis of the QCHAT-10

**DOI:** 10.1038/s41598-025-26131-9

**Published:** 2025-11-07

**Authors:** Lydia J. Sollis, Dennis P. Wall, Peter Y. Washington

**Affiliations:** 1 Department of Information and Computer Sciences, University of Hawaii at Manoa, Honolulu, HI 96822 USA; 2 Department of Epidemiology, University of Hawaii Cancer Center, Honolulu, HI 96813 USA; 3 Department of Pediatrics (Clinical Informatics), Stanford University, Stanford, CA 94305 USA; 4 Department of Biomedical Data Science, Stanford University, Stanford, CA 94305 USA; 5 Department of Psychiatry and Behavioral Sciences (by courtesy), Stanford University, Stanford, CA 94305 USA; 6 Division of Clinical Informatics and Digital Transformation, Department of Medicine, University of California-San Francisco (UCSF), San Francisco, CA 94143 USA

**Keywords:** Autism spectrum disorder (ASD, or autism), Machine learning, QCHAT-10, Early intervention, Diagnostic assessment, Feature importance, Paediatric research, Autism spectrum disorders, Mathematics and computing

## Abstract

Early identification improves life outcomes for individuals with autism. This study addresses a central question: do compact subsets of the most predictive QCHAT-10 items, when fed into machine learning (ML) models trained to reproduce the full questionnaire’s screening result, generalize to predicting clinician-established autism diagnoses in independent clinical settings? We applied ML to the 10-question QCHAT-10, training models on New Zealand (*n* = 1054) and Saudi Arabian (*n* = 506) datasets with QCHAT-derived labels and testing on Polish data with clinical diagnoses (*n* = 252). Recursive Feature Elimination identified four-item models retaining three common features: eye contact, following gaze direction, and pretend play. When tested on clinically-diagnosed Polish cases at the 0.3 prediction threshold, the New Zealand model achieved an AUROC of 85% ± 13 (sensitivity 91%, specificity 50%), while the Saudi model reached 87% ± 11 (sensitivity 84%, specificity 80%), compared to the Polish four-item model’s cross-validation AUROC of 91% ± 5. These findings demonstrate partial transfer from the prediction of assessment scores to clinical diagnosis. The convergence on eye contact, gaze following, and pretend play suggests these behaviors represent robust autism risk markers. Compact assessment tools offer advantages, including reduced burden, shortened administration, and simplified deployment, with direct applications for targeted digital phenotyping.

**Supplementary Information:**

The online version contains supplementary material available at 10.1038/s41598-025-26131-9.

## Introduction

Autism Spectrum Disorder (ASD, or autism) is a complex condition characterized by varied developmental impacts that can lead to social, communication, and behavioral challenges. The global prevalence is approximately 1 in 100 children^1^, though likely underestimated given the US prevalence is 1 in 31 for 8-year-olds as of 2022^2^. Early intervention, beginning as young as 2 or 3 years, can significantly enhance long-term outcomes and increase access to resources^3^.

However, autism diagnosis is notoriously time-consuming, relying on detailed examinations of developmental history and behaviors^4,5^. This delays crucial early treatments. The United States Preventive Services Task Force advocated for universal autism screening in 2016, leading to the integration of tools such as the Ages and Stages Questionnaires (ASQ) and the Modified Checklist for Autism in Toddlers (M-CHAT) into routine pediatric visits^6^. Despite these tools, implementation faces significant hurdles due to time constraints and workflow disruptions^7^. Additionally, most autism research has been conducted in westernized, English-speaking countries^8^, raising questions about tool validity across various settings.

### Machine learning applications to diagnostic instruments

ML has been applied to two categories of autism assessment: (1) formal diagnostic instruments and (2) early detection questionnaires. One prominent diagnostic tool is Canvas Dx, developed by the health technology company Cognoa, which became the first authorized diagnostic system for autism. Cognoa’s initial research found that 7 of 93 Autism Diagnostic Interview-Revised (ADI-R) items were sufficient to classify autism with 99.9% accuracy^9^, and ten or fewer of the 29 ADOS items achieved 97% accuracy^10–12^. Prospective validation studies between 2012 and 2017^13–16^ culminated in a final study incorporating a caregiver questionnaire, video analysis of smartphone-uploaded videos, and a healthcare provider questionnaire. This multimodal approach outperformed baseline assessment tools by 35% for AUC and 69% for specificity at 90% sensitivity^17^. The finalized Canvas Dx tool yielded a positive predictive value (PPV) of 81%, a negative predictive value (NPV) of 98%, and 98% sensitivity^18^, with a median positive diagnosis age more than a year younger than the US overall^19^. In 2023, algorithmic threshold optimization improved Canvas Dx’s ability to provide determinate outputs without altering its accuracy or intended use^20^.

### Machine learning applications to questionnaires

In parallel with diagnostic applications, extensive research has focused on applying ML to questionnaires to identify children who may benefit from a comprehensive diagnostic evaluation. ML algorithms can assist in feature reduction by identifying the most predictive indicators of autism^21^ and enable the incorporation of data modalities such as video, which could screen at-risk children outside traditional healthcare settings and fast-track them for further diagnostic evaluation^7,20,22–25^.

Several recent studies have demonstrated ML’s potential for feature reduction. Küpper et al. identified five predictive ADOS features, yielding an AUROC of 87%^26^. Washington et al. achieved 92% AUROC using a single Social Responsiveness Scale question, and found consistency with three of Duda et al.’s top six features distinguishing autism from ADHD^27^. Tariq et al. tested whether a reduced set of questionnaire-derived features could be used in conjunction with non-expert raters watching 3-minute home videos of US children^22^. A subsequent study applied this technique to videos of Bangladeshi children, demonstrating the utility of ML tools in developing countries where clinical resources are scarce^23^. In 2021, Washington et al. explored the effect of privacy-preserving methods on model performance using the same set of reduced features, concluding that sensitivity was preserved (96.0%), while specificity (80.0%) and accuracy (88.0%) remained at acceptable levels^24^. Another study concluded that feature replacement methods could compensate for low video quality in video-based assessments^25^. Using recursive feature elimination on an Italian full-length Q-CHAT with 25 questions, Tartarisco et al. obtained an AUROC of 95%, utilizing 14 questions^28^.

In 2017, Thabtah published widely used Q-CHAT-10 datasets collected in New Zealand using the AutismTests mobile application^29^. Subsequent studies achieved high performance, with Vakadkar et al. achieving an accuracy of 97% and an F1 score of 98%^30^ on data pooled from three distinct age groups, while another study saw AUROC values of 98% and 97% for male and female versions of sex-segregated models with 10-fold cross-validation^31^. In 2022, Thabtah explored the use of a Self-Organizing Map (SOM) to independently derive class labels using feature clustering. Performance on the SOM-refined dataset was significantly higher.

More recently, Rahman and Subashini used deep neural networks (DNNs) on the QCHAT dataset. They trained two separate classifiers, one on Polish Toddlers’ Q-CHAT data and the New Zealand (NZ) Q-CHAT-10 datasets, achieving high sensitivity, specificity, and AUC scores of 100%/99%/100% for QCHAT-10 and 93%/83%/97% for QCHAT, respectively^33^.

### Applications in varying geographical and clinical contexts

Despite extensive literature on Q-CHAT and Q-CHAT-10 questionnaire datasets, studies rarely test models on independent datasets collected in different settings^34^. A critical limitation of studies using these datasets is the lack of clinical validation. Class labels are derived from assessment scores rather than confirmed diagnoses, meaning models predict whether children score above thresholds, not whether they have a clinical autism diagnosis. This circular validation may inflate apparent accuracy and limit clinical applicability.

Validation across heterogeneous settings faces challenges beyond language translation^35–37^. Psychometric properties vary significantly based on sample characteristics, including socioeconomic status, parental education, and contextual behavior intepretations^38,39^. Differences in eye contact norms, pretend play expectations, or gesture use may affect parental questionnaire responses^40,41^. Additionally, referral patterns and diagnostic pathways to diagnosis differ substantially across healthcare systems, potentially resulting in different populations of children receiving assessments versus diagnostic evaluations^42,43^. These factors suggest that tools optimized in one context may not maintain predictive validity elsewhere, highlighting the need for external validation studies^44^.

To address this, we trained models separately using NZ Q-CHAT-10 data from Thabtah et al.^45^ and Saudi Arabian QCHAT-10 data from Kaggle^46^. We used stratified k-fold cross-validation to select the best-performing model, examined feature importance, and employed recursive feature elimination. Each model was validated on the other dataset to enhance sensitivity. A final evaluation tested both models on the Polish QCHAT dataset^47^ with the original and adjusted cutoffs models, and compared results with Polish-trained model cross-validation outcomes. This approach addresses a fundamental question: do compact subsets of the most predictive QCHAT-10 items, trained to reproduce screening results, generalize to predicting clinician-established autism diagnoses in an independent clinical setting? This question is vital because compact screening instruments offer concrete deployment advantages: reduced caregiver burden, shortened administration time, and simplified translation. These are critical factors for scaling autism assessment globally. Moreover, compact, reproducible feature sets translate directly into digital phenotyping design specifications. If joint attention, repetitive behaviors, and social reciprocity drive predictive performance, developers can target audio-visual and interaction sensors that quantify those specific behavioral constructs, streamlining data collection while preserving diagnostic signal.

## Methods

### Study overview

**Fig. 1:**
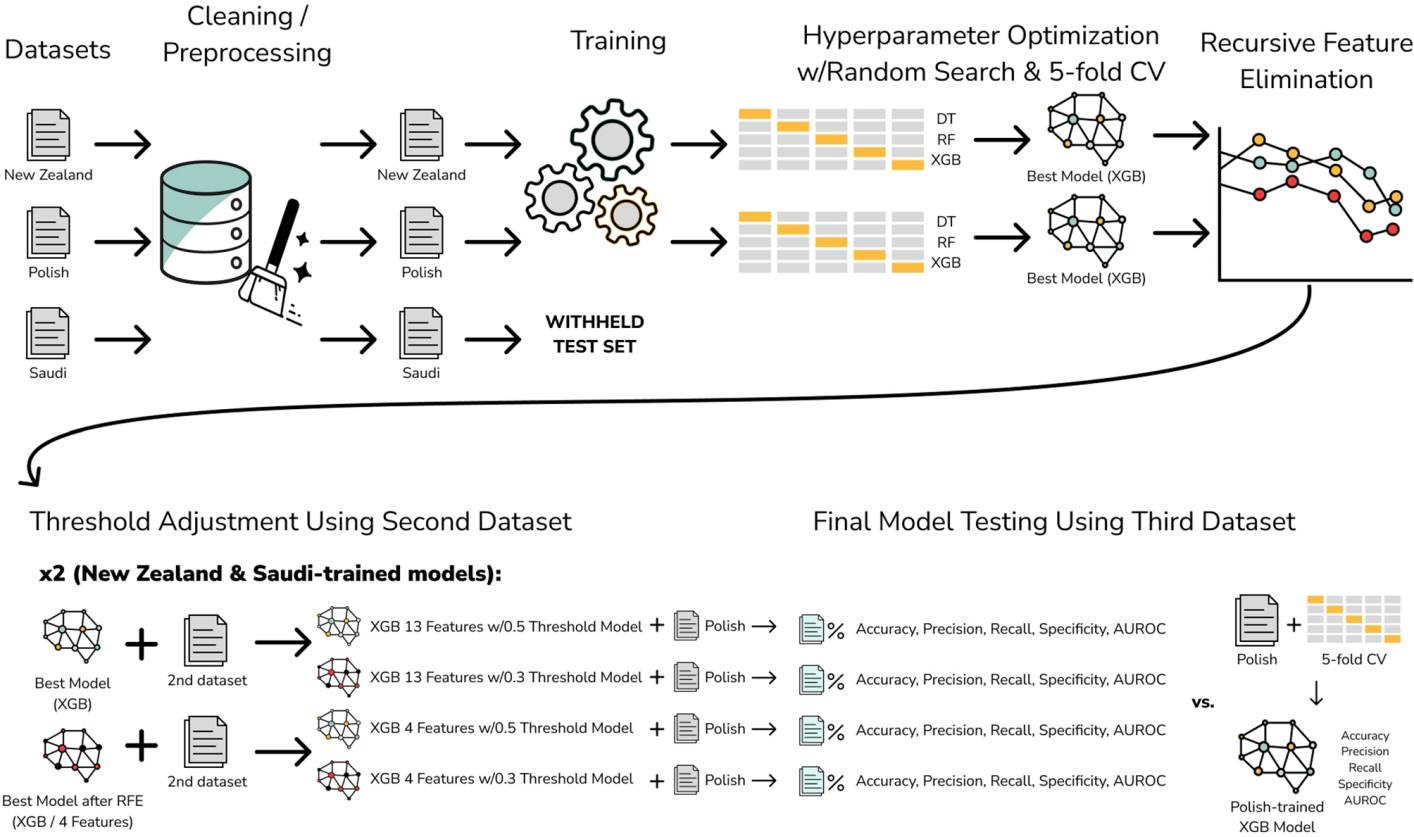
Pipeline for model training and testing. Three distinct datasets were cleaned and preprocessed. The NZ and Saudi datasets were then used to train DT, RF, and XGB models, utilizing Randomized Search and 5-fold cross-validation to determine optimal hyperparameters. The best model from each dataset was refined further using recursive feature elimination. Subsequently, both the full-featured and refined models were validated on the other dataset, and the threshold for positive prediction was adjusted to optimize sensitivity while maintaining acceptable balanced accuracy and specificity values. Finally, both models were tested on the Polish dataset, and the results were compared to cross-validation performance on the Polish data.

**Fig. 2:**
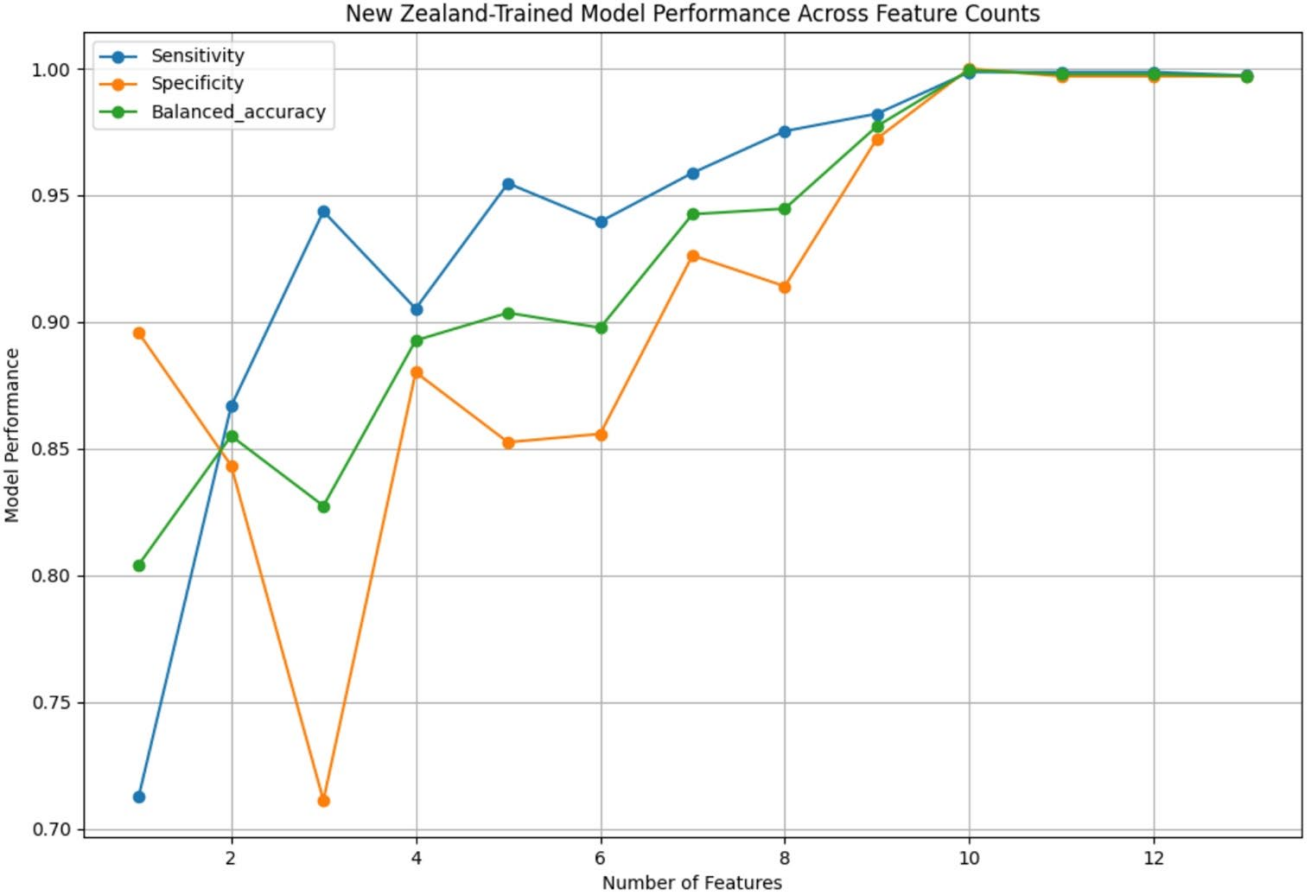
Performance across feature counts for XGB model trained on NZ dataset.

Our central procedure consisted of (1) training and hyperparameter optimization on the Saudi and the NZ datasets (Fig. 1), (2) choosing optimal prediction thresholds using the opposing dataset for validation, (3) evaluating both models on the Polish data that contains official diagnostic labels (i.e., not using the QCHAT-10), and (4) comparing results against the Polish dataset’s cross-validation performance.

It is important to note that this study evaluates label-source/construct shift. The NZ and Saudi datasets utilize class labels derived from QCHAT-10 scores (where greater than three is considered positive), whereas the Polish dataset contains labels from comprehensive clinical diagnoses (i.e., using ADOS-2, ADI-R, and DSM-5 criteria). Models trained on reduced feature sets of the NZ and Saudi data learn to predict assessment outcomes when using the full QCHAT-10. The critical question is whether these assessment-score predictors transfer to predicting clinician-established diagnoses, representing both label-source shift (from screening score to clinical diagnosis) and a construct shift (from questionnaire threshold to comprehensive diagnostic evaluation), in addition to potential recruitment and population differences.

We preprocessed the datasets for consistent feature encoding, then employed stratified k-fold cross-validation during model training to maintain balanced representation of positive and negative cases. Next, we tested Decision Tree, Random Forest, and XGBoost models with randomized hyperparameter search, analyzed feature importance, performed recursive feature elimination, and adjusted the probability threshold for positive prediction to maximize sensitivity while maintaining acceptable specificity. For our final evaluation, we tested the optimized models on the Polish dataset to validate their applicability to clinical diagnosis and robustness in diverse demographic and environmental contexts.

### Model inputs and outputs

XGB model inputs consist of the ten QCHAT-10 questions plus demographic data such as the child’s sex, age in months, and familial developmental disorder history. Responses are binarized: for questions 1–9, ‘Sometimes,’ ‘Rarely,’ or ‘Never’ is assigned a value of 1, indicating a lack of developmentally appropriate behaviors. Conversely, for question 10, responses of ‘Always,’ ‘Usually,’ or ‘Sometimes’ are assigned a 1, reflecting a behavior more likely to occur in autistic children. The QCHAT-10 questions are listed in Supplementary Table S1. Cumulative scores of greater than three suggest a positive result^48^.

Model output is a binary indicator (positive or negative assessment). We train and validate two models on datasets with labels derived from assessment scores (Saudi and NZ datasets), predicting whether a child’s QCHAT-10 responses will sum to a score of greater than three. This represents a circular prediction task where questionnaire items predict questionnaire-derived outcomes, which is acceptable given that we seek to identify a minimal set of features that predict the final score. Final testing on an external validation set involves using the Polish dataset with labels derived from comprehensive clinical diagnoses using instruments beyond the QCHAT-10.

### Datasets

We use three datasets sourced from different countries, all with participant ages ranging from 12 to 36 months (Supplementary Table S2). Welch’s t-test and the Mann-Whitney U test showed no significant age difference between positive-scoring and negative-scoring children.

***Q-CHAT data from NZ (diagnostic labels derived from Q-CHAT).*** This dataset includes 1054 entries collected by Dr. Fadi Fayez Thabtah. It was collected via the ASDTests assessment application, a mobile tool allowing individuals to complete the Q-CHAT-10 and ASD-10 questionnaires in 11 languages^29,49–51^. Class values are automatically assigned based on the a threshold score of four or higher. The mean age of children in this dataset is 28 months (SD = 8.0).

***Q-CHAT data from Saudi Arabia (diagnostic labels derived from Q-CHAT).*** This dataset was retrieved from Kaggle. The dataset consists of 506 entries, with 341 classified as having a positive assessment result for autism and 165 as negative^47^. The questionnaire consisted of an Arabic-translated version of the Q-CHAT-10 collected online via Google Forms; however, other details about recruitment are unspecified. Labels were assigned based on an assessment score greater than three. The mean age of children was 24 months (SD = 8.3).

***Q-CHAT data from Poland (diagnostic labels derived separately from Q-CHAT assessment using official diagnostic criteria).*** This dataset features full-length Q-CHAT results from 252 Polish toddlers, including 135 diagnosed with autism and 118 who are normally developing^47^. Participants were recruited from specialist diagnostic centers in Warsaw, with class labels based on diagnoses confirmed through comprehensive clinical assessment, including ADOS-2 (Autism Diagnostic Observation Schedule-2), ADI-R (Autism Diagnostic Interview-Revised), and clinical judgment in accordance with DSM-5 criteria. The mean age was 21 months (SD = 2.1).

### Models

We selected tree-based models for their interpretable structure and ability to learn simple decision rules from data features, beginning with a Decision Tree^52^ and progressing to their more complex extensions, Random Forest^53^ and XGBoost (XGB)^54^. Decision Trees are interpretable ML models that make predictions by learning simple decision rules from data features. Random Forests improve upon single trees by creating multiple decision trees and aggregating their predictions, while XGB is an advanced ensemble method that builds trees sequentially. Each tree corrects errors of prior trees, using gradient boosting with regularization to achieve high accuracy while preventing overfitting.

### Feature encoding and pre-processing

The NZ dataset comprises 1054 examples with no missing values. It includes responses to the QCHAT-10 questionnaire, the overall questionnaire score, and demographic information such as age in months, sex, and family history of developmental disorder, among others; it also records whether the child was born with jaundice and who completed the test (self, family member, etc.). Although the NZ and Saudi datasets share identical features based on QCHAT-10 data, the Polish dataset, derived from QCHAT-25 data, includes only sex, family history of developmental disorder, and age in months as common demographic features; it also includes 25 questions, ten of which correspond to the QCHAT-10. It incorporates additional features such as child ID, whether the child was preterm, birth weight in grams, mother’s education, and whether the child had siblings with autism.

During preprocessing, the QCHAT scores used to determine class labels were omitted. The ten questions and three common demographic features were retained across datasets. No transformations or imputations were needed, given complete data. Feature scaling was deemed unnecessary for tree-based models.

### Model evaluation

Our analysis followed three steps: first, training one model on each dataset (NZ and Saudi) using cross-validation for parameter selection; second, validating on the other assessment dataset to determine probability thresholds maximizing sensitivity empirically; and third, testing both threshold settings on Polish clinical diagnosis data.

For initial model evaluation, we tested DT, RF, and XGBoost models on the NZ and Saudi datasets, and XGBoost only on the Polish dataset. Stratified k-fold cross-validation was employed using k = 5 folds and 80/20 validation splits. The NZ dataset used 843 samples for training and 211 for validation per fold; the Saudi dataset used approximately 405/101 per fold; and the Polish dataset (for cross-validation comparison) used 202/50 per fold. This method ensured that each subset contained a balanced representation of both positive- and negative-scoring participants.

Hyperparameter optimization used RandomizedSearchCV, with a minimum of 500 candidates per dataset-model combination. The hyperparameter search space is detailed in Supplementary Table S3, and optimal hyperparameters selected are in Supplementary Table S4. Models were evaluated on accuracy, precision, sensitivity, specificity, and ROC-AUC; the best-performing model from each dataset was selected based on the highest metrics across the majority of these measures. Supplementary Table S2 details metrics for the best-performing models.

After hyperparameter optimization, final models were retrained on complete datasets (1054 for NZ, 506 for Saudi) and tested on the Polish dataset (*n* = 252: 135 with confirmed autism diagnoses, 117 typically developing). Recursive feature elimination for NZ and Saudi models halted at four features for both.

Both models were validated using thresholds of 0.3 and 0.5 for positive predictions, with the opposing dataset serving as the validation set. Finally, each model was tested on Polish data, and the resulting accuracy, precision, sensitivity, specificity, and AUROC values were compared to those from 5-fold cross-validation on the Polish dataset.

## Results

### Feature importance

Supplementary Table S5 displays the feature importances for XGBoost, the top-performing model, on each dataset. Features related to QCHAT-10 responses were generally more important than demographic features, likely because class labels were derived directly from assessment scores in 2 out of 3 datasets. Individual question importance varied widely across datasets.

Question 9 (“Does your child use simple gestures (e.g., wave goodbye)?”) ranked highly across both Saudi and NZ datasets, with a feature importance of 0.24 in the NZ model (ranked 1st) and 0.19 in the Saudi model (ranked 2nd). Both NZ and Polish models ranked question 6 (“Does your child follow where you’re looking?”) in the top five features, scoring 0.10 for NZ and 0.21 for Polish.

Beyond these commonalities, the models had few similarities in their top five most predictive features. In the Saudi model, the top three features were almost equally predictive, with question 6 (“Does your child follow where you’re looking?”) scoring 0.21, question 9 scoring 0.19, and question 2 scoring 0.18. The next most predictive question had a score of only 0.07. The Polish model had a similar pattern, with question 3 (“Does your child point to indicate that s/he wants something (e.g., a toy that is out of reach)?”) scoring 0.22, and question 4 (“Does your child point to share interest with you (e.g., pointing at an interesting sight)?”) scoring 0.20, followed by a sharper drop-off for question 5 (scoring 0.14). For the NZ model, question 9 scored 0.24, with the next most predictive question being only half as impactful (question 7 with a score of 0.12), and question 1 (“Does your child look at you when you call his/her name?”) scored 0.11.

Demographic features such as family history of PDD, age in months, and sex were less important. Age in months ranked 12th of 13 features for NZ and 13th for both the Polish and Saudi-trained models. Sex ranked 13th for NZ and 12th for the other two models. Family history of PDD ranked 11th for NZ, 7th in the Polish-trained model, and 10th in the Saudi model. Significantly, because the class variables for the NZ and Saudi models were based on survey responses to the QCHAT questions, it is expected that the demographic variables will be less predictive. However, sex, age, and family history of PDD were similarly less predictive for the Polish model. Family history of PDD, the most predictive demographic characteristic for the Polish model, was similarly predictive to the other models, with a feature importance score of 0.03 (compared to 0.04 for the Saudi model and 0.02 for the NZ model).

### Recursive feature elimination

**Fig. 3:**
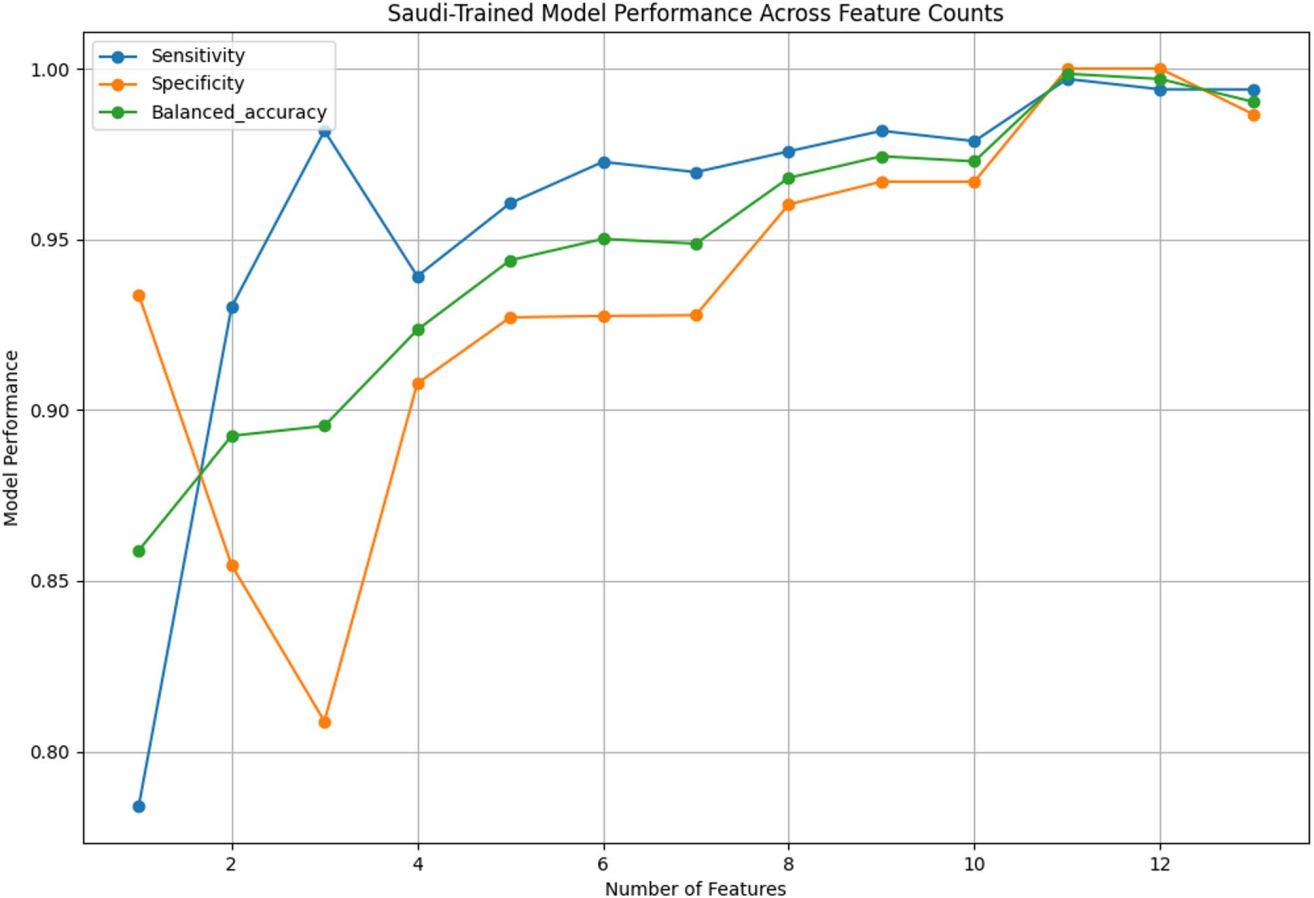
Performance across feature counts for the XGB model trained on the Saudi dataset.

Recursive Feature Elimination (RFE) was performed on the best-performing model for each dataset (see Fig. 2 for the NZ-trained model and Fig. 3 for the Saudi-trained model). The models with the best metrics were then compared with those trained on all features, yielding two models for each of the NZ and Saudi datasets. Supplementary Table S6 summarizes the number of features and performance metrics of each model. For the NZ model, all features except questions 5, 6, 7, and 9 were eliminated, while the Saudi model retained questions 2, 5, 6, and 9.

Notably, both the NZ and Saudi models maintained metrics of 88% or higher after Recursive Feature Elimination (RFE). The NZ model achieved a balanced accuracy of 89%, sensitivity of 91%, specificity of 88%, and an AUROC of 95% with only four features. The Saudi model performed slightly better, with corresponding values of 92%, 94%, 91%, and 98%.

Both models retained the following questions as features after RFE: question 5 (“Does your child pretend (e.g., care for dolls, talk on a toy phone)?”), question 6 (“Does your child follow where you’re looking?”), and question 9 (“Does your child use simple gestures, e.g., wave goodbye?”). This retention suggests these features have universal significance. Additionally, question 6 ranked 5th among the most important features for the NZ model (0.10 importance score) and the most important for the Saudi model (0.21 importance score), underscoring its relevance. Conversely, questions 2 and 5 did not consistently exhibit high predictive value in feature importance rankings.

### Threshold adjustment to maximize sensitivity

Maximizing sensitivity in an assessment tool can have significant public health benefits. Early detection and intervention for autistic children can lead to better health outcomes, including improved developmental trajectories and access to appropriate support services. While optimizing for sensitivity may lead to lower specificity, it can be an acceptable trade-off in these contexts. As a result of this goal, the probability threshold for a positive prediction was adjusted to maximize sensitivity while maintaining a specificity of greater than 0.5. The NZ models used the Saudi dataset for the threshold adjustment step, and vice versa. The 4-feature models obtained by RFE were included in the analysis. Results are displayed in Table 1.

**Table 1:** Cross-dataset validation for threshold optimization.

Train dataset	NZ dataset		Saudi dataset	
Hyperparameter optimization dataset	Saudi dataset (4 features)		NZ dataset (4 features)	
Threshold	0.5	0.3	0.5	0.3
Balanced accuracy	0.91 ± 0.01	0.84 ± 0.02	0.86 ± 0.01	0.79 ± 0.01
Sensitivity	0.91 ± 0.01	0.96 ± 0.01	0.90 ± 0.01	0.98 ± 0.00
Specificity	0.91 ± 0.01	0.71 ± 0.02	0.83 ± 0.01	0.59 ± 0.02
AUROC	0.96 ± 0.03	0.96 ± 0.03	0.95 ± 0.04	0.95 ± 0.04

For both the 4-feature and 13-feature models, the optimal threshold for a positive prediction was adjusted from 0.5 to 0.3 to enhance sensitivity values while maintaining reasonable precision and specificity. The NZ-trained model achieved sensitivity and specificity values of 0.91 ± 0.01 before adjustment, with adjustment increasing sensitivity to 0.96 ± 0.01 but with lower specificity values. The Saudi model began with sensitivity/specificity values of 0.90 ± 0.01 and 0.83 ± 0.01, respectively, and a specificity of 0.98 ± 0.00 and a specificity of 0.59 ± 0.02 post-adjustment.

### Testing on clinical diagnosis data (polish dataset)

After validation and threshold adjustment, the 4-feature models were tested on the Polish dataset, which had not been utilized for training or validation. The results are displayed in Table 2.

For the NZ 4-feature model test on Polish clinical diagnosis data, we observed severe trade-offs between sensitivity and specificity at each threshold. At the 0.5 cutoff, sensitivity was 79% ± 3, while specificity was 80% ± 3. Adjusting to the threshold 0.3 yielded a high sensitivity of 0.91 ± 2, but specificity dropped to an unacceptably low 0.50 ± 3.

The Saudi 4-feature achieved better balance when tested on the Polish clinical diagnosis data. At the 0.5 threshold, sensitivity was 74% ± 3 and specificity was 85% ± 2. Adjusting to the 0.3 threshold increased sensitivity to to 84% ± 2 while maintaining reasonable specificity of 80% ± 3.

**Table 2:** Held out test set results of NZ and Saudi models on Polish datasets.

Train dataset	NZ dataset		Saudi dataset	
Threshold adjustment dataset	Saudi dataset		NZ dataset	
Held-out test dataset	Polish dataset (4 features)		Polish dataset (4 features)	
Threshold	0.5	0.3	0.5	0.3
Balanced accuracy	0.80 ± 0.03	0.70 ± 0.03	0.79 ± 0.03	0.82 ± 0.02
Sensitivity	0.79 ± 0.03	0.91 ± 0.02	0.74 ± 0.03	0.84 ± 0.02
Specificity	0.80 ± 0.03	0.50 ± 0.03	0.85 ± 0.02	0.80 ± 0.03
AUROC	0.85 ± 0.13	0.85 ± 0.13	0.87 ± 0.11	0.87 ± 0.11

### Comparison with polish model 5-fold cross-validation

Lastly, we compared metrics obtained by testing the trained models on the Polish dataset (Table 2) with those from 5-fold cross-validation of the Polish dataset with an XGB model (Table 3). The Polish 4-feature model achieved an AUROC of 91% ± 9 with a sensitivity of 0.84 ± 0.09 and specificity of 90% ± 5 with both the 0.3 and 0.5 cutoff thresholds.

**Table 3:** Results of 5-fold cross-validation on XGB model with Polish dataset.

Dataset	Polish dataset (4 features)	
Threshold	0.3	0.5
Balanced Accuracy	0.87 ± 0.06	0.87 ± 0.06
Sensitivity	0.84 ± 0.09	0.84 ± 0.09
Specificity	0.90 ± 0.05	0.90 ± 0.05
AUROC	0.91 ± 0.05	0.91 ± 0.05

## Discussion

This study addressed a fundamental question: can compact QCHAT-10 subsets, trained to reproduce screening scores, generalize to predicting clinician-established diagnoses in independent clinical settings? Our results indicate partial success with important caveats.

### Label transfer performance

Four-item compact models trained on assessment-score data transferred to clinical diagnosis prediction with moderate success. The Saudi model maintained reasonable performance (AUROC 87 ± 11%, 84% sensitivity, 80% specificity at 0.3 threshold), approaching the benchmark from direct training on Polish clinical data (AUROC 91 ± 5%). However, the NZ model showed substantial specificity degradation (50%) despite high sensitivity (91%).

Notably, both training procedures independently identified three common features: eye contact (question 2), pretend play (question 5), and gaze following (question 6). This convergence suggests that these behaviors may represent robust autism risk markers^55^.

### Clinical applicability

The clinical applicability of our 4-item abbreviated questionnaires warrants careful consideration. While both models retained questions about eye contact, pretend play, and gaze following—suggesting potential universal markers—the dramatic reduction in specificity for the NZ 4-item model (50% at 0.3 threshold) limits its practical utility. The Saudi 4-item model performed better, maintaining 80% specificity at the 0.3 threshold when tested on Polish data. This compares favorably to previous attempts at questionnaire reduction: Allison et al. created the 10-item QCHAT from the original 25-item version^56^, while our results suggest further reduction to 4 items may be feasible within specific geographic contexts but not as universal assessment tools. Previous ML-based assessment reductions have shown similar patterns, with Wall et al. achieving high accuracy with reduced item sets within single populations^10^. For clinical implementation, we recommend using the full QCHAT-10 until environment-specific validation of abbreviated versions can be conducted using clinically diagnosed samples.

All three datasets included children aged 12–36 months with comparable mean ages, and no significant age differences were observed between positive and negative groups within any dataset. This age homogeneity within datasets suggests that age did not confound assessment results. However, the absence of age-stratified analyses remains a limitation, as autism symptoms emerge and manifest differently throughout toddlerhood. The QCHAT-10’s performance may vary across this 24-month developmental span, and future studies should consider age-stratified analyses or narrower age bands to optimize assessment accuracy at specific developmental stages.

### Limitations

This study has several limitations. First, our fundamental design involves source/construct shift. Models were trained to predict whether Q-CHAT-10 scores exceeded a threshold, then tested on clinical diagnoses. Second, we used datasets that differed considerably in recruitment context and size (though this may also be considered an advantage of this study). The Polish dataset (*n* = 252) represents a clinical sample from specialist diagnostic centers, while the NZ dataset (*n* = 1054) was collected through a freely available mobile app, likely attracting participants with existing concerns, and the Saudi dataset’s (*n* = 506) recruitment methods remain unknown. High positive-screen rates (69% in NZ and 67% in Saudi Arabia, compared to 54% in the Polish clinical sample) far exceed the population prevalence of autism, suggesting non-representative samples rather than general population representation. These sampling differences limit our ability to assess true generalizability and may inflate performance metrics when applied to populations with a higher pre-test autism probability.

Third, training on assessment scores rather than confirmed diagnoses may limit the models’ ability to accurately distinguish between autistic and neurotypical individuals. The absence of Saudi dataset recruitment details, combined with limited information about the NZ dataset’s self-selected sample, represents a significant limitation in interpreting model generalizability.

Fourth, the importance of individual questions varied across models despite commonalities in RFE-retained features (eye contact, gaze following, and pretend play). Developing universally applicable assessment tools may require identifying consistently predictive features across varied populations. Threshold adjustment to maximize sensitivity universally resulted in lower specificity during testing, often unnecessarily, as some models exhibited high sensitivity at test time even without threshold adjustments. Future work should optimize thresholds to balance sensitivity and specificity effectively. Additionally, all datasets included children aged 12–36 months with no significant age differences between groups, suggesting age did not confound results. However, the absence of age-stratified analyses remains a limitation, as autism symptoms manifest differently throughout toddlerhood.

## Conclusions

Looking forward, this study demonstrates that compact screening instruments trained on assessment scores can partially transfer to predicting clinical diagnoses, though with notable performance degradation. The convergence on eye contact, gaze following, and pretend play across independent training sets suggests these behaviors may represent robust markers for autism risk. However, inconsistent transfer success, varying recruitment contexts, potential sampling biases, and the fundamental label-source shift from screening scores to clinical diagnoses represent significant limitations that necessitate further investigation.

Future research should (1) obtain clinical diagnosis labels for multiple datasets to enable true cross-setting generalization testing, (2) examine whether specific training data characteristics, such as population characteristics, screening instrument properties, or clinical setting, predict successful transfer of diagnostic labels, (3) develop hybrid approaches combining screening instruments with other modalities, such as video analysis or digital phenotyping to improve specificity while maintaining sensitivity, and (4) conduct prospective validation studies deploying compact instruments in real-world clinical workflows to assess their practicality as triage tools.

## Supplementary Information

Below is the link to the electronic supplementary material.

Supplementary Material 1

## Data Availability

The datasets used and analyzed during the current study are available from the following sources:1. **New Zealand QCHAT-10 Dataset** : The autism screening data for toddlers collected by Dr. Fadi Fayez Thabtah is available from the ASDTests screening application repository. The dataset can be accessed at [ASDTests Repository] (https://example-repository-link.com) 0.2. **Polish QCHAT-10 Dataset** : The dataset featuring QCHAT scores from Polish toddlers is publicly accessible and can be found at Mendeley data, [data.mendeley.com/datasets/tmpkt2mfkg/2] (https://data.mendeley.com/datasets/tmpkt2mfkg/2) 0.3. **Saudi Arabia QCHAT-10 Dataset** : This dataset was obtained from Kaggle and is publicly accessible. It can be downloaded from [kaggle.com/datasets/asdpredictioninsaudi/asd-screening-data-for-toddlers-in-saudi-arabia](https://www.kaggle.com/datasets/asdpredictioninsaudi/asd-screening-data-for-toddlers-in-saudi-arabia) .All datasets include the minimal data necessary to interpret, replicate, and build upon the findings reported in this article. Any additional information required can be requested from the corresponding author, Peter Washington at [peter.washington@ucsf.edu](mailto: peter.washington@ucsf.edu) .The code repository associated with this study is located at: https://github.com/ucsfdigitalhealth/qchat-10-ml-analysis.

## Abbreviations

ADI-R: Autism diagnostic interview-revised
ADOS-2: Autism diagnostic observation schedule, second edition
ASD: Autism spectrum disorder
AUROC: Area under the receiver operating characteristic curve
DT: Decision tree
ML: Machine learning
NPV: Negative predictive value
PDD: Pervasive developmental disorder
PPV: Positive predictive value
QCHAT-10: Quantitative checklist for autism in toddlers (10-item)
RF: Random forest
RFE: Recursive feature elimination
XGB/XGBoost: Extreme gradient boosting

## Key machine learning terms

Cross-validation: Testing model performance by training on part of the data and testing on another part, repeated multiple times. We used this only for hyperparameter optimization and for comparing Polish model performance
Features: Individual pieces of information used for predictions (e.g., questionnaire responses, age, sex)
Labels/class values: The outcome being predicted—“autism-positive assessment” (QCHAT-10 score ≥ 3) or “no autism indicators” (score < 3); for the Polish dataset, labels represent clinical autism diagnosis
Model: A mathematical formula learned from data that predicts outcomes based on input features
Recursive feature elimination (RFE): Creating shorter questionnaires by systematically removing less important questions while maintaining accuracy
Sensitivity: Proportion of true positive cases correctly identified (minimizing missed cases)
Specificity: Proportion of true negative cases correctly identified (minimizing false alarms)
Threshold adjustment: Modifying the cutoff point for positive predictions to increase sensitivity (catch more cases) at the cost of reduced specificity (more false positives)
Training: Teaching the model to recognize patterns using examples with known outcomes
