# Supplementary material for: Compact subsets of autism screening items predict clinical diagnoses with a machine learning analysis of the QCHAT-10
Source: Sci Rep. 2025 Nov 7;15:39091. doi: 10.1038/s41598-025-26131-9 (PMC12594979; doi:10.1038/s41598-025-26131-9)
Supplement: Supplementary file 1 — Supplementary Material 1 [file 41598_2025_26131_MOESM1_ESM.docx]

**Supplementary Information**

**Table S1.** List of questions included on the QCHAT-10.

| **Feature** | **Question** |
| --- | --- |
| A1 | Does your child look at you when you call his/her name? |
| A2 | How easy is it for you to get eye contact with your child? |
| A3 | Does your child point to indicate that s/he wants something (e.g., a toy that is out of reach)? |
| A4 | Does your child point to share interest with you (e.g., pointing at an interesting sight)? |
| A5 | Does your child pretend (e.g., care for dolls, talk on a toy phone)? |
| A6 | Does your child follow where you’re looking? |
| A7 | If you or someone else in the family is visibly upset, does your child show signs of wanting to comfort them (e.g., stroking their hair, hugging them)? |
| A8 | Would you describe your child's first words as (typical)? |
| A9 | Does your child use simple gestures (e.g., wave goodbye)? |
| A10 | Does your child stare at nothing with no apparent purpose? |

**Table S2.** Summary of the characteristics of each dataset included in the study.

| **Dataset** | **Columns** | **Number of examples** | **Sex Ratio (M: F)** | **Age in Months** | **% Class Distribution (ASD: NT)** |
| --- | --- | --- | --- | --- | --- |
| NZ | Child ID; Age in months; Sex; Ethnicity; Jaundice; Family member with ASD; Who completed the test; Class/ASD Traits (Yes/No); Questions related to ASD traits (A1 to A10); Q-CHAT-10-Score (out of 10) | 1054 | 70:30 | Mean: 27.9  SD: 8.0  Range: 12-36 | 69:31 |
| Polish | Child ID; Age in months; Sex; Class (ASD/Normal); Pre-term; Birth-weight; Siblings (Yes/No); Number of siblings; Mothers education; Sibling with ASD (Yes/No); Sum Q-CHAT (out of 100); 25 Questions related to ASD traits | 252 | 62:38 | Mean: 21.1  SD: 2.1  Range: 18-24 | 54:46 |
| Saudi Arabia | Child ID; Age in months; Sex; Family member with ASD; Who completed the test; Class/ASD Traits (Yes/No); Questions related to ASD traits (A1 to A10); Q-CHAT-10-Score (out of 10); Region/Province | 506 (481 after duplicate removal) | 31:69 | Mean: 24.3  SD: 8.3  Range: 12-36 | 67:33 |

**Table S3**. Hyperparameter search space for initial model training.

| **Dataset** | **Model** | **Hyperparameter Search Space** |
| --- | --- | --- |
| NZ | Decision Tree | criterion: gini, entropy  max depth: 10 to 50  min samples split: 2 to 10  min samples leaf: 1 to 4  max features: None, sqrt, log2 |
|  | Random Forest | n estimators: 100 to 400  criterion: gini, entropy  max depth: 10 to 70  min samples split: 2 to 10  min samples leaf: 1 to 4  max features: None, sqrt, log2  bootstrap: True, False |
|  | XGBoost | n estimators: 100 to 400  max depth: 3 to 10  learning rate: 0.01 to 0.3  subsample: 0.5 to 1  col sample by tree: 0.5 to 1  gamma: 0 to 0.3  reg lambda: 1 to 3  reg alpha: 0 to 0.2 |
| Saudi | Decision Tree | criterion: gini, entropy  max depth: 10 to 50  min samples split: 2 to 10  min samples leaf: 1 to 4  max features: None, sqrt, log2 |
|  | Random Forest | n estimators: 50 to 400  criterion: gini, entropy  max depth: None, 5 to 50  min samples split: 2 to 10  min samples leaf: 1 to 4  max features: None, sqrt, log2  bootstrap: True, False |
|  | XGBoost | n estimators: 100 to 400  max depth: 3 to 10  learning rate: 0.01 to 0.3  subsample: 0.5 to 1  col sample by tree: 0.5 to 1  gamma: 0 to 0.3  reg lambda: 1 to 3  reg alpha: 0 to 0.2 |
| Polish | XGBoost | n estimators: 100 to 400  max depth: 1 to 10  learning rate: 0.01 to 0.3  subsample: 0.5 to 1  col sample by tree: 0.5 to 1  gamma: 0 to 0.5  reg lambda: 1 to 5  reg alpha: 0 to 0.2 |

**Table S4.** Best model performance and hyperparameters on each dataset.

| **Dataset** | **Model** | **Model Hyperparameters** | **Metrics** |
| --- | --- | --- | --- |
| NZ | XGBoost | n estimators: 300  max depth: 10  learning rate: 0.3  subsample: 0.8  col sample by tree: 0.5  gamma: 0.3  reg lambda: 3  reg alpha: 0.1  use label encoder: False  eval metric: logloss | ROC-AUC: 1.00 ± 0.00  Accuracy: 1.00 ± 0.00  Precision: 1.00 ± 0.00  Sensitivity: 1.00 ± 0.01  Specificity: 1.00 ± 0.01 |
| Saudi | XGBoost | n estimators: 300  max depth: 7  learning rate: 0.1  subsample: 0.5  col sample by tree: 0.7  gamma: 0  reg lambda: 3  reg alpha: 0.1  use label encoder: False  eval metric: logloss | ROC-AUC: 1.00 ± 0.00  Accuracy: 1.00 ± 0.00  Precision: 1.00 ± 0.00  Sensitivity: 1.00 ± 0.01  Specificity: 1.00 ± 0.00 |
| Polish | XGBoost (only model tested) | n estimators: 400  max depth: 1  learning rate: 0.3  subsample: 1  col sample by tree: 1  gamma: 0.5  reg lambda: 2  reg alpha: 0  use label encoder: False  eval metric: logloss | ROC-AUC: 0.94 ± 0.05  Accuracy: 0.88 ± 0.03  Precision: 0.90 ± 0.02  Sensitivity: 0.87 ± 0.06  Specificity: 0.89 ± 0.02 |

**Table S5.** Feature importances for top performing models (both XGBoost).

| **Feature Importance Ranking** | **NZ XGB Model** | | **Saudi XGB Model** | | **Polish XGB Model** | |
| --- | --- | --- | --- | --- | --- | --- |
|  | Feature Name | Feature Importance | Feature Name | Feature Importance | Feature Name | Feature Importance |
| 1 | A9_Score | 0.24 | A6_Score | 0.21 | A3_Score | 0.22 |
| 2 | A7_Score | 0.12 | A9_Score | 0.19 | A4_Score | 0.20 |
| 3 | A1_Score | 0.11 | A2_Score | 0.18 | A5_Score | 0.14 |
| 4 | A5_Score | 0.10 | A8_Score | 0.07 | A1_Score | 0.09 |
| 5 | A6_Score | 0.10 | A4_Score | 0.06 | A7_Score | 0.09 |
| 6 | A2_Score | 0.08 | A5_Score | 0.06 | A2_Score | 0.09 |
| 7 | A4_Score | 0.08 | A3_Score | 0.05 | A10_Score | 0.05 |
| 8 | A3_Score | 0.05 | A1_Score | 0.04 | family_pdd | 0.03 |
| 9 | A8_Score | 0.04 | A7_Score | 0.04 | A6_Score | 0.02 |
| 10 | A10_Score | 0.04 | family_pdd | 0.04 | A9_Score | 0.02 |
| 11 | family_pdd | 0.02 | A10_Score | 0.03 | A8_Score | 0.01 |
| 12 | age_months | 0.02 | sex | 0.02 | sex | 0.01 |
| 13 | sex | 0.01 | age_months | 0.01 | age_months | 0.01 |

**Table S6.** Evaluation metrics and retained most-predictive features after performing RFE on XGB models trained on Saudi and NZ datasets.

|  | NZ Dataset | Saudi Dataset |
| --- | --- | --- |
| Features Retained | A5_Score, A6_Score, A7_Score, A9_Score | A2_Score, A5_Score, A6_Score, A9_Score |
| Balanced Accuracy | 0.89 ± 0.03 | 0.92 ± 0.01 |
| Sensitivity | 0.91 ± 0.01 | 0.94 ± 0.02 |
| Specificity | 0.88 ± 0.05 | 0.91 ± 0.04 |
| AUROC | 0.95 ± 0.02 | 0.98 ± 0.01 |
